# Agrochemicals Use Practices and Health Challenges of Smallholder Farmers in Ghana

**DOI:** 10.1177/11786302211043033

**Published:** 2021-09-17

**Authors:** Suleyman M Demi, Suzanne R Sicchia

**Affiliations:** Department of Health and Society, University of Toronto Scarborough, Toronto, ON, Canada

**Keywords:** Agrochemicals, health, smallholder farmers, Ghana

## Abstract

**Background:**

Globally, Africa is one of the continents that uses the lowest quantity of agrochemicals in farming. However, unsafe chemical use practices are high among farmers in Africa, posing serious health and environmental consequences. This study seeks to address three questions: (1) What factors motivate/compel smallholder farmers in Ghana to use agrochemicals? (2) What safety precautions or unsafe chemical use practices can be found in the communities? (3) What are the health implications of agrochemical use among smallholder farmers in Ghana?

**Methodology:**

The study used purposive and simple random sampling techniques to select 136 individuals for the survey, out of which 31 individuals were eliminated, and 105 participants were selected for in-depth interviews and focus group discussions. Additionally, participants’ observations were collected, workshops were facilitated, and documents analyses were conducted. Qualitative data were analyzed using NVivo software and the quantitative data were analyzed using SPSS version 23.0.

**Findings:**

The study found that environmental challenges, activities of NGOs, government policy, lack of or high cost of labor, and competition among farmers were major factors influencing farmers’ decisions to use agrochemicals. Present agrochemical use in Ghana poses a risk to health and the environment. Finally, the study discovered chemical poisoning and low self-reported health quality as major health implications of agrochemical use in the communities.

## Introduction

Globally, Africa is one of the continents that uses the lowest quantity of agrochemicals (agrochemicals are used in this paper to collectively refer to synthetic chemicals including pesticides and fertilizers) in farming. This can be attributed to several factors, including the subsistent nature of farming where most farmers grow crops mainly to feed themselves and only sell the surplus, inability to purchase agrochemicals due to fewer financial resources, low return on investments, and so on.^1,2^ Africa accounts for less than 5% of the global pesticides market and only 2% to 4% of usage rate.^3,4^ This figure excludes the agrochemicals donated to African countries through philanthro-capitalist organizations spearheading the green revolution. Despite the reduced use of agrochemicals, unsafe chemical use practices are high among farmers in Africa.^4,5^ Many factors account for this phenomenon, including small farm sizes, which compel farmers to come into direct contact with agrochemicals, and the normalization of agrochemicals by smallholder farmers due to limited knowledge of safety precautions. For instance, the average farm size in the United States and Canada are 421 and 778 acres respectively^6^ compared to 4.9 acres in Ghana^7-9^ and several African countries.^10^

Most African farmers have limited economic incentives and financial resources to afford sophisticated farm machinery to apply agrochemicals; hence they resort to the use of manual/simple farm tools such as knapsack sprayers, buckets, brushes, and brooms in the application of agrochemicals.^11-13^ Furthermore, many farmers apply pesticides without the appropriate Personal Protective Equipment (PPE) such as nose mask, overall coats, goggles, gloves, Wellington boots, etc., which further exposes them to chemical poisoning with grievous health and environmental consequences.^14,15^ According to Northern Presbyterian Agriculture Services and Partners (NPASP)^27^ of Ghana, 15 farmers died because of chemical poisoning in the Upper East region of Ghana. A study in Uganda, for example, found that 99.5% of the smallholder farmers applied pesticides without PPE and 92.7% mixed chemicals with their bare hands.^15^ Deaths linked to the unsafe use of agrochemicals have also been reported in both Rwanda and Burundi.^16^ However, the use of agrochemicals in subsistence farming is increasing in Africa,^17,18^ which is orchestrated by continuous promotion of agrochemicals in Africa by corporations.^19,20^ Onwona-Kwakey et al^21^ found that food crop farmers in Ghana used agrochemicals at a rate of 1.3 to 13 times higher than the recommended doses. Overdose application of pesticide is also reported among vegetable farmers in Tanzania^22^ and among smallholder farmers in Ghana.^13^

In Ghana, the most widely used agrochemicals are fertilizers and pesticides. Farmers apply various fertilizers such as NPK, Muriate of Potash (MOP), Urea, Ammonium Sulfate (AS), Single Super Phosphate (SSP), and Triple Super Phosphate (TSP) to their crops.^69,75^ Atrazine, Roundup, Adwumawura, Sunphosate, Kondem, Ceresate, Chemosate, Stam F34, Gramoxone are among the commonly used herbicides by farmers.^12,27,51,69^ Furthermore, various insecticides such as Karate, Diazinon, Cydim super, Dursban 4E, Dimethoate etcetera are used by farmers to control insect pests.^51,74^ Farmers use these insecticides to reduce the incidence of crop damage or crop failure. Agrochemicals such as Karate, Diazinon, and Sumithion are among the recommended insecticides used for vegetable farming. However, farmers sometimes use non-recommended insecticides including Polytrine, Delphos, Thiodan, Thionex, Cypercal, Dursban, Fastac etcetera on vegetables.^74^

Ghana is traditionally one of the African countries that relies less on agrochemicals^7-9^; however, the situation is changing rapidly as agrochemicals are becoming a mainstay of agriculture in Ghana. For example, pesticide use in Ghana has increased from 9% in 1991 to 47% in 2003.^23^ This astronomical increase creates serious health and environmental consequences.^11,24^ Data from the Environmental Protection Agency (EPA) in Ghana indicates 540 varieties of chemicals have been registered for use in agriculture and public health.^21,25^ Studies show between 70% and 85% of farmers in Ghana use agrochemicals^1,2,21^ and usage is high among vegetable and cash crops farmers.^11,21^ The increased use of agrochemicals by farmers with limited knowledge in safety precautions and lack of Personal Protective Equipment (PPE) raise concerns among health experts and the general public. There is a call for the relevant authorities to monitor the impact of agrochemicals used in Ghana.^26^ In Ghana, regulations govern the use of agrochemicals; however, implementations and monitoring are limited due to lack of staff and logistics.^27^

Several other factors, including the emergence of new pests and diseases, a desire for higher yields, larger farm sizes, and environmental challenges account for increased use of agrochemicals.^2,21,28^ That said, agrochemical use depends on specific geographical location, individual farmers’ decisions, and the ability and accessibility of the agrochemicals. It is reported that pest and disease outbreak can decrease crop yield by 40%.^15^ Conversely, appropriate application of agrochemicals can increase crop yields significantly,^29,30^ a return that encourages more farmers to use agrochemicals. However, the question remains to what extent agrochemical uses impact the environment and humans, and whether the gain in yield outweighs the health and environmental damage. These questions are beyond the scope of the paper but worth considering in future studies. Studies show the most proven and cost-effective way of enhancing soil fertility and protecting cash crops against pest and disease infestation include the use of organic fertilizers and cultural practices such as pruning, regular weed clearing, and farm sanitation^31^ which are the core of Indigenous farming systems.

There is extensive literature focusing on field testing or a review of accessible agrochemicals.^32,33^ Others investigated farmers’ behavior and perception on agrochemical use^34-37^ and some explored the potential of biological pest control.^38,39^ Few studies that investigated the determinants of pesticides used in Ghana focused on cash crops such as cocoa and rice^12,31,67^ with little attention to food crop farmers. Secondly, studies on the effects of agrochemicals are overwhelmingly quantitative, missing the voices of smallholder farmers who are disproportionately affected. Consequently, the overarching goal of the study is to assess agrochemical use practices among smallholder farmers in Ghana. Specifically, this study seeks to address 3 research questions: (1) What factors motivate/compel smallholder farmers in Ghana to use agrochemicals? (2) What safety precautions or unsafe chemical use practices can be found in the communities? (3) What are the health implications of agrochemical use on the smallholder farmers in Ghana? This study is significant as it addresses agrochemicals use practices in Ghana from farmers’ perspectives. The findings of this study could help formulate policies to promote environmental health and food safety among growers and the general population. The next section describes the methodology of the study.

## Methodology

**Figure 1.**
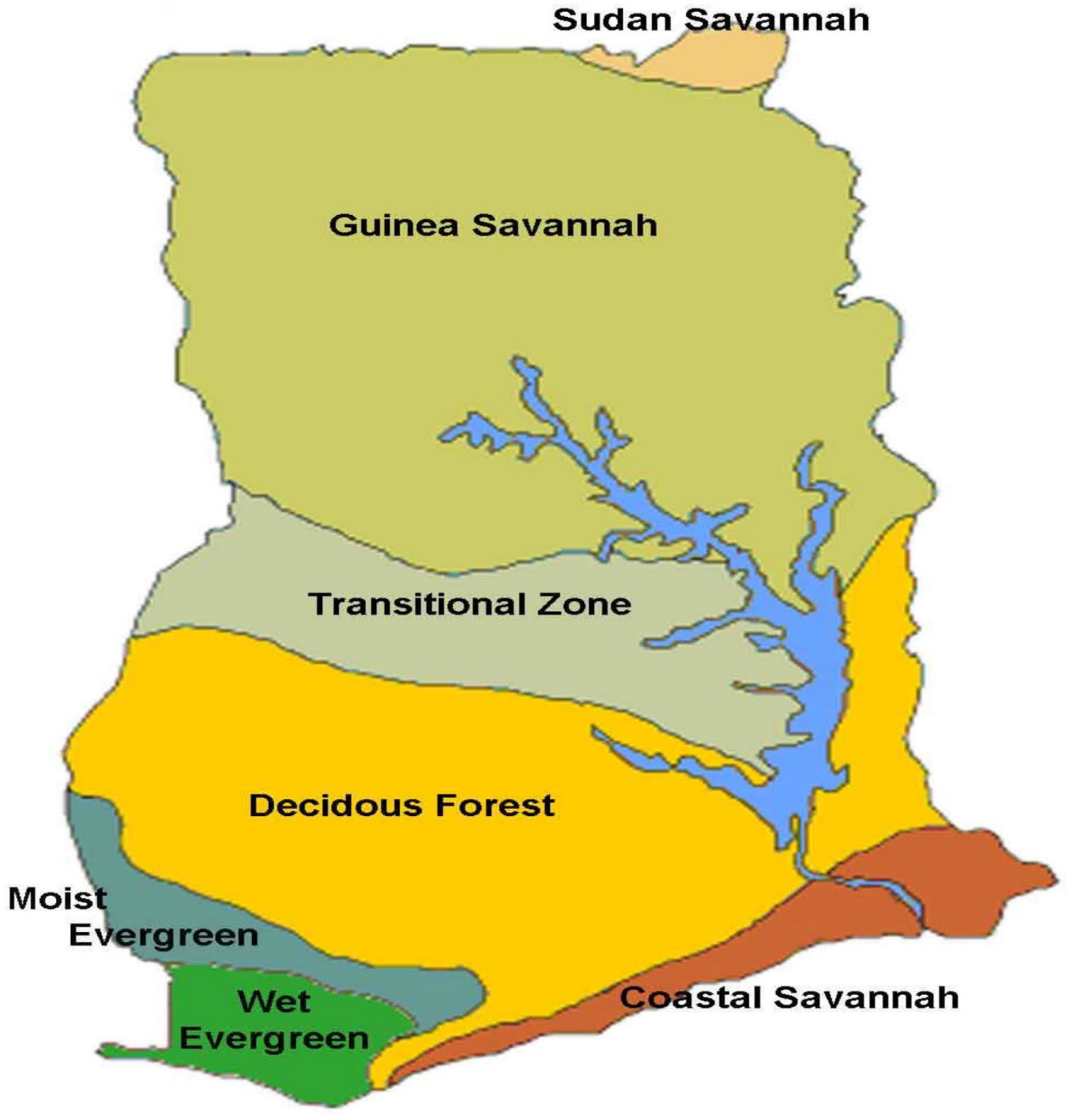
Map of agro-ecological zones in Ghana. Source: FAO.

This study was conducted in Ghana, located in the western part of Africa, from March to June 2017. Ghana is bordered by Burkina Faso on the north, Ivory Coast on the west, Togo on the east, and the Atlantic Ocean to the south. The majority (82.5%) of the rural population are smallholder farmers^40^ (Ghana Statistical Service, GSS, 2014). Ghana has 7 agro-ecological zones: Sudan Savannah, Guinea Savannah, Transitional Zone, Deciduous Forest, Moist Evergreen, Wet Evergreen, and Coastal Savannah as shown in Figure 1.

These agro-ecological zones can be broadly categorized into 3 belts: Middle belt, representing the transitional zone; Northern belt, zones above the transitional zone; and the Southern belt, zones below the transitional zone. Multistage sampling techniques were used to select 3 agro-ecological zones, 3 regions, 3 districts, and six communities in which the sampled population was drawn. The first stage involved the purposive selection of 3 agro-ecological zones: Guinea savanna, Transition zone, and Coastal savanna in which the regions and districts were selected. The second stage involved the selection of 1 region each in 3 of the agro-ecological zones: Northern Region (NR) representing Guinea Savana, Bono Region (BR) representing Transitional zone and Greater Accra Region (GAR) representing Coastal Savana. The third stage involved a selection of 1 district/municipality in each of the 3 regions. Tolon district was selected from NR, Wenchi Municipality was selected from the BR, and Ga West Municipality was selected from GAR. Two communities were selected in each of the districts. The names of communities are Wantugu and Tolon representing Tolon district of the NR, Amponsahkrom and Ayigbe representing Wenchi Municipality of the BR and Deideiman area and Otsirkomfo representing Ga West Municipality of the GAR.

Tolon district was selected because it is one of the districts in the Northern Region severely affected by climate change. The resulting dry weather conditions raise important questions about how climate change influences agrochemical use in these communities. Tolon is also the host district for the Nyankpala campus of the University for Development Studies (UDS) dedicated to the study of agriculture, and so offered possible insights on the impact research in the university has on food production practices of local farmers. Further, the selection of Tolon district allowed the researcher to sample the views of some of the students and faculty on food production practices in the district. Ga West Municipality was selected because it was one of the few districts in GAR chosen for the presidential initiative on cassava in 2001, because of its viable agricultural activities.^41,42^ Wenchi Municipality located in BR was selected because of its mixture of forest and drylands, therefore it serves as a bridge between the Northern belt which is mostly drylands and the Southern belt that consists of coastal and forest lands.

The final stage of the sampling was the use of purposive and simple random sampling to select 136 individuals for the survey out of which 31 individuals were eliminated, and 105 participants were selected for an in-depth interview and focus group discussion. The study used purposive sampling in most cases to enable the researchers to select individuals who are knowledgeable on the subject under investigation. Furthermore, a simple random sample was also used to capture other perspectives and knowledgeable but shy individuals. The study eliminated 31 participants due to incomplete and short responses that lack additional information from the already gathered data. The criteria for selecting the individual research participants included being a smallholder food crop farmer with considerable years of farming experience or a professional in the food-related field located within the study area.

The farmers selected were food crop farmers who grow crops mainly to feed themselves and sell the surplus. In addition to farming, the female farmers engage in off-farm activities such as selling cooked food, indigenous leafy vegetables, firewood, and farm products in the community and the local market. The male farmers engage in trading and rearing small ruminants (ie, goat and sheep) and poultry including chicken, guinea fowls, etc., to supplement their income. The major food crops farmers cultivate include maize, cassava, cocoyam, green beans, and vegetable such as garden eggs, tomatoes, onion, pepper, okra, cabbage, etc., mostly among smallholder farmers in the South and Middle belt. However, the farmers in the Northern belt also grow rice, cassava, groundnut, beans, potatoes, and to a lesser extent yam and millet as the soils are unfavorable to the cultivation of yam and millet.

The composition of the participants is as follows: 56 smallholder farmers (26 women and 30 men), 5 students, and 5 agricultural educators (ie, teachers and extension officers), 1 representative of the Peasant Farmers Association of Ghana (PFAG), 1 representative from the Food and Agriculture Organization, Ghana (FAO), and 2 senior officers from the Ministry of Food and Agriculture (MoFA). Additionally, a total of 48 smallholder farmers participated in 5 focus group discussions out of which 13 were among the participants who took part in the in-depth interview. The focus groups were further used to validate responses from individual participants. Some of the questions asked include: What are some of the crops do you grow in this community? What types of agrochemicals do you use? When and how do you apply agrochemicals? What are some of the effects or challenges do you experience with the use of fertilizers and pesticides?

In accordance with prior works by Freeman^43^ and Muijeen et al^44^ the focus groups were composed of between 6 and 12 smallholder farmers. The sample size was similar to the work of Appiah-Opoku^45^ and Bhattarai et al^46^ who interviewed between 45 and 150 individuals for a mixed-method study. The University of Toronto ethics board approved this study. Purposive sampling was used in most cases because it enabled the researcher to select individuals based on the possession of characteristics of interest.^47,48^ Also, the researchers conducted workshops, participant observation, and analysis of relevant documents from the Ministry of Food and Agriculture (MoFA) and Ministry of Health (MoH) to supplement and triangulate data from in-depth interviews, focus growing discussions, and surveys. Survey data were analyzed using SPSS 23.0, while data from in-depth interviews, focus groups, and participant observation were analyzed through coding and thematization using NVivo software. All quotes in this paper are suffix with abbreviation such as WS representing workshop, FG representing focus group discussion, EO representing extension officer, and Id representing in-depth interview. This paper focuses solely on the qualitative data to complement already extensive quantitative studies on agrochemical use.

## Findings

The study found that the majority (85%) of the farmers are moving away from Indigenous farming systems which rely on low input farming to the use of agrochemicals. This shift in practice from Indigenous methods to the use of agrochemical compromises their health and environmental sustainability as most farmers lack Western education and are therefore limited in terms of their understanding and ability to abide by the safety precautions. These findings corroborate findings of earlier studies^49-51^ which discovered that most vegetable farmers in Ghana used pesticides to control pests and diseases on their farm. It further confirms the assertion that farmers in developing countries rely on herbicides to avoid weed competition and labor constraints.^52^ The current studies, however, found 5 key factors influencing farmers’ decision to use agrochemicals: environmental challenges, activities of NGOs, government policy, lack of or high cost of labor, and competition among farmers. This finding is different from a study by Anang and Amikuzuno,^49^ who discovered that factors including farm size, farm income, mechanization, extension contact, and distance to the source of pesticide and production systems, influence the decision of rice farmers in Northern Ghana to use agrochemicals. However, this difference may be attributed to the fact that the current study is relatively broader in scope and captures smallholder farmers from different geographical regions.

## Factors Influencing Farmers to Use Agrochemicals

This section discusses the 5 key factors that motivate or compel farmers to use agrochemicals starting with environmental challenges.

### Environmental challenges

One major factor that compels farmers to use synthetic agrochemicals is worsening environmental conditions link to climate change. This problem is compounded by the new varieties of crops that farmers are growing which are susceptible to diseases and pests, and therefore, necessitate the use of agrochemicals. There is ongoing promotion of short duration varieties of crops and associated agrochemicals in sub-Saharan Africa.^19,20^ Farmers are responding and increasing their use of agrochemicals despite serious repercussions. In one of the community workshops, farmers expressed concerns with the use of agrochemicals but attributed their use of agrochemicals to the worsening environmental conditions:

We are aware that the fertilizers and chemicals are not good, but we don’t have a choice. The soils are so depleted that if I go to farm, nothing will come out for me and my family if I don’t apply fertilizer. It means that year there will be a hunger for me and my family. So, in other [sic] to get something to support my family, I have to go and apply fertilizer so that I can get something. So even the chemicals, I am aware, sometimes when you are spraying, and it touches your skin, you see the effects of the chemical. So is not that we don’t know. (WS3)

The quote shows the extent to which farmers have become dependent to the use of agrochemicals. It further indicates farmers are gradually moving away from Indigenous methods of soil management practices depended on for centuries in response to worsening environmental conditions. Ultimately, farmers have experienced the negative effects of agrochemicals and amenable to explore sustainable farming practices. The finding confirms similar study by Obiri et al,^69^ who found that 100% of farmers agreed that herbicides negatively affect soil fauna and flora. The sentiment expressed in the quote is sadly the persistent theme across most of the interviews conducted in the North. Several farmers were categorical that they cannot produce food without the use of agrochemicals: “Here we have no choice, we have no option, is the fertilizer that we have been using because, I don’t have the energy to carry the manure to the farm” (Fuseina, IdI). In addition to Fuseina, the majority of the participants argued if they farm without agrochemicals, they will experience total crop failure. However, Fuseina’s quote suggests farm manure can help address some of the crisis even though she indicated their options are limited. In this way, lack of energy to convey organic manure to the farm and scarcity of organic manure can be seen to contribute to over-dependency on agrochemicals. Taken together, these quotes reveal an important cultural shift among smallholder farmers toward agricultural intensification at the expense of indigenous farming practices as also noted by several studies.^5,11,12^

There is a sense of disappointment and frustration within a segment of the academic community about the excessive use of agrochemicals in the study areas, especially in the North.^1,12^ Interaction with a senior faculty member elicited critical questions: Does your work look at agrochemical use among smallholder farmers? Did the farmers tell you they use agrochemicals? Can they read and write? If they cannot read and write how are they able to apply the agrochemical per the recommendation? Farmers agreed in principle with the senior faculty’s assertion by pointing out their limitations in comprehending safety precautions of some of the chemicals they used. For instance, farmers were amazed to hear that some of the chemicals they used such as DDT or ammonium sulfate are banned substances due to their toxicity or increased soil acidity. (Notably, DDT is officially banned in Ghana but NPASP^27^ has reported traces of DDT found in some products [ie, milk] which is an indication of DDT being smuggled into Ghana. Also farmers referred to any chemical that kills pests as DDT) Their amazement is captured in this quote:

It is so annoying that you people (Westerners), you know this thing is not good, you banned it, you don’t use it, and you know very well that when you come to this side of the country a lot of us have no formal education, we can’t read, we can’t write, we will not be able to know that this particular chemical is banned, so now, where are they putting us? Are they not going to kill us? (WS3)

The quote above indicates the farmers are unable to identify banned chemicals even if they hear of them because they cannot read the labels and know the active ingredient of the chemicals. The finding is aligned with several studies in Ghana which point to the farmers’ failure to identify active ingredients, composition, and brand of agrochemicals they applied to their crops.^1,5,11,12,70^ Similarly, a cross-sectional study in Uganda found that farmers had limited knowledge about agrochemicals and most of them did not use PPEs.^71^ However, the frustration of farmers also indicates their willingness to change their practices if they have an alternative to agrochemical use.

### Activities of NGOs

The study found that some farmers in the remote communities were introduced to synthetic agrochemicals by NGOs working in their communities. The result confirms earlier studies which found deliberate promotion of agrochemicals in Africa.^4,19,20^ This situation is relatively higher in the Northern belt of Ghana compared to South and Middle belts. This can be attributed to the large number of NGOs operating in the Northern part of Ghana probably due to elevated temperatures that make the conditions unfavorable to smallholder farmers.^53,54^ According to farmers, NGOs introduced them to short-duration varieties, which are bred to adapt to synthetic agrochemicals. For instance, Iddi, an elderly male farmer who also rears cattle in addition to crop cultivation in the Northern belt explains:

. . .is of late that they introduced moringa, ADRA introduces it to us. It was the same ADRA who gave us the three months’ varieties of maize. . . when NGOs started trickling in into this community especially ADRA, ADRA introduced us to some technologies, row planting and how to apply fertilizers. And we moved from the small, small bags we were getting to about 22 bags. (IdI)

NGOs present themselves as saviors to the farmers and many of their practices provide immediate benefits such as higher yield and income on the short run, so farmers are easily convinced. The government of Ghana also recognizes NGOs as development partners,^7,8^ hence, this has empowered them to operate without thorough scrutiny. There is a proliferation of NGOs in Northern Ghana and the majority failed to submit their budget for scrutiny.^53,54^ Although some of the activities of NGOs provided relief to farmers, they ultimately undermined Indigenous food systems and contributed to the extinction of Indigenous food crops and in so doing created long-term havoc to farming communities. Indeed, in one of the community workshops, farmers complained about being unable to grow their indigenous crops:

. . .we used to grow late millet, we used to grow Bambara beans, and we used to grow some types of cowpea, and all that, which were very helpful to us. But now if you look at the soil now, they are so depleted that the soil doesn’t support the cultivation of these Indigenous crops. (WS2)

When farmers were asked what impedes their cultivation of Indigenous crops listed in the quote, they responded that the crops grow tall, stalky, and produce no grains. However, farmers’ association of vegetative growth of crops to nutrient deficiency is quite the opposite especially when crops listed are legumes. Crops grow vegetatively when there is excessive nitrogen in the soil rather than nutrient deficiency. Some farmers got it right: “. . .if you come to this community, because of too many chemicals we don’t produce yam again, because yam will not do at all on the soil, apart from cassava, I think that is because of the abuse of these chemicals that has caused the problem” (WS2). The assertion of farmers was validated by other farmers in different communities and MoFA officials.

However, unlike the Northern belt where farmers cannot produce crops without the use of agrochemicals, farmers in the Southern and Middle belts are not experiencing the same crisis. This study found that chemical usage in the South and Middle belt are higher among vegetable farmers than staple crop farmers confirming previous studies.^1,5,11^ The senior faculty informant, a professor of agronomy and communication, contends that farmers are motivated to use agrochemicals by NGOs with a vested interest:

There are organic ways of pest control that are very effective, but farmers are not using them because some of the NGOs just want to push the sale of agrochemicals. They use huge sums of money for advertisement convincing farmers to use agrochemicals. . . There are some chemicals that when you spray or apply to crops you must wait for some months before you can consume. How will the farmers get to know this information when they cannot read and write? In a serious country, even the chemicals they use to store maize or crops after the four months, somebody must go there and analyze to determine the residual effects, but nobody is doing that. (SF, IdI)

Another faculty informant agreed with this senior faculty about the way activities of NGOs are making farmers and their soil dependent to agrochemicals: “We have propagated the use of chemicals such that farmers don’t want to go back to traditional means of farming. . .if you look at our indigenous way of farming like use of compost manure or green manuring that is the best” (Shaibu). The frustrations of these academics are well placed because their claims were confirmed by farmers during 1-on-1-interviews and focus group discussions.

### Government policies

Government policies play a major role in directing farmers’ activities. Government policies aim at helping farmers to address environment challenges, provide subsidies to support farmers, and ensure food security, among other impacts. For instance, EU countries spend €54 billion annually since 2006 under Common Agricultural Policies (CAP) to support farmers.^55^ In 2008, the government of Ghana introduced a fertilizer subsidy program where the government absorbs 40% to 50% of the cost of fertilizers to help boost smallholder farmers’ productivity.^7,8^ The fertilizer subsidy program was initiated to boost food production to ensure food security.^7,8^ Furthermore, it was in response to similar policies introduced in neighboring countries to prevent smallholder farmers in Ghana from being disadvantaged. However, this policy resulted in unintended consequences such as the smuggling of the subsidized fertilizers to neighboring countries to sell, and increased use of pesticides. According to extension officers, the fertilizer subsidy program normalized the use of agrochemicals to the extent that farmers ignore the consequences or do not believe the effects of agrochemicals:

. . .we are educating farmers on the need to grow organic foods due to the chemical concentration, if the government has any incentives to give farmers. What is the government doing? They are reducing the price of chemicals we are preaching against. So, the government is motivating them to use more chemicals. (EO4)

The above quote indicates the government fertilizer subsidy programme is a major factor motivating smallholder farmers to use agrochemicals because it makes the fertilizers cheaper and gives credibility to fertilizer use. Huge sums of money are spent annually on the fertilizer subsidy. In 2017, for instance, the government of Ghana allocated US$4 million for the purchase of pesticides and the education of farmers to address armyworm invasion.^2^ The question lingering in the minds of many farmers is: Why is the government promoting the use of agrochemicals if they are not good? However, some farmers believe that not every farmer has access to the fertilizer subsidy program. Additionally, the program does not extend to pesticides. Consequently, these farmers call on the government to drastically reduce the prices of agrochemicals to make them affordable to them. The study observed that farmers who call for a reduction in the prices of agrochemicals are mostly in the south. This can be attributed to the favorable environmental conditions in the South, which result in a higher return on investment. Again, farmers in the South do not face harsh environmental conditions as their counterparts in the North. Farmers in the Northern and to some extent in the Middle belt rather call on the government to find a substitute to the synthetic fertilizers to address health and environmental challenges.

### Lack of labor or high cost of labor

The study discovered that labor scarcity and the high cost of labor also influences farmers’ decisions to use synthetic agrochemicals. The finding confirms Wumbei et al^70^ who found that yam farmers used herbicides to avoid prohibitive labor cost. However, the situation is relatively more prominent in the Southern belt compared to the Northern and Middle belt. Farmers in the Southern and Middle belts mostly use herbicides and insecticides due to labor scarcity while their counterparts in the Northern belt mostly use fertilizers. Also, tractor services are rare in the South compared to the Northern belt due to the topography of the land, which is characterized by mountains, making many of the farms inaccessible to the tractor. Secondly, urbanization in the communities is resulting in many lands demarcated as plots of land for building houses and owners of the plots prohibit the use of a tractor to clear weeds on the plots. Thirdly, there is a dwindling number of farmers as many people are moving away from farming into the labor market due to scarcity of land; hence, there is no need for the District Department of Agriculture to keep farm implements such as tractors. Farmers who traditionally rely on farm laborers to clear their farmlands no longer get laborers as the youth continue to troop to the cities in search of white-collar jobs due to the proximity of the communities to the nation’s capital, Accra. Farmers, especially female farmers, now solely rely on herbicides to clear their farmlands. Alice, a divorcee who takes care of her nieces and nephews explains:

It is very difficult to get a labourer to clear your farm. When the labourers lack money and you mention to them that you have a land that needed to be cleared. . . the person will force you to get the money and promise you to go to the farm first thing in the morning but alas, you will follow the person for two months and the person will still be deceiving you. . . We don’t have many labourers here, because of that when we buy round-up (herbicide) and others then we spray the weeds. Me for instance, when I fix the knapsack at my back, I can spray one acre. (IdI)

However, most farmers are untrained on the chemical application; therefore, health precautions are compromised in their practices posing major health and environmental threats.^4,56^ Also, it is worth noting that the active ingredient of round-up used by Alice is glyphosate which was found to be carcinogenic in the United States.^72,73^ However, most farmers are not aware of the danger of the chemicals they apply to their crops. The knapsack sprayer as used by Alice is also prone to leakage, thus, increasing the risk of farmers exposure to the harmful chemicals.^11,57^

### Competition among farmers/modern farming

The study found that competition among smallholder farmers influences their decision to use agrichemicals. For instance, unlike Western countries where there are premium prices for organic foods and farmers are given incentives by the government for sustainably producing food^58,59^ such incentives are lacking or not common in developing countries such as Ghana. Consequently, farmers who are engaging in organic farming are disadvantaged and ridiculed by their colleagues for not practicing a modern type of farming. This compels some smallholder farmers who otherwise would have stuck to organic farming to follow their counterparts who use chemicals. This was revealed by Otumi, a fisherman who later became a food crops farmer:

So, if you decide to do organic farming and someone who uses chemicals compares his/her farm produce to yours he will notice vast difference/result and the person will say you are not a good farmer. That is the issue, so it has made us abandon our traditional way of farming. Traditional farming would have helped us but now you can’t go back to traditional farming, you can’t get the traditional seeds. (IdI)

Otumi emphasized that hybrid crops are bred to adapt to chemicals, therefore refusing to apply agrochemicals results in lower yield. Hence, farmers who do not use agrochemicals are likely to be criticized by their colleagues for not being good farmers. This sentiment was expressed by a significant number of farmers. Otumi’s comment raises the issue of how farmers are losing their Indigenous seeds, which according to some farmers better adapt to their climatic conditions. The extinction of Indigenous seeds is a major political issue that affects farmers especially with the introduction of GMOs in Ghana.^60,61^ Additionally, organic farmers in Ghana receive lower prices for their farm products or risk their product being rejected because consumers consider the bigger and fresher products as healthier. This was revealed by one of the research participants:

And the market women, if you go to Agbobloshie [food market in Accra] like this, I frequently see them, the hoteliers, and the students when they come to buy cabbage, tomatoes, onion, etc. they select the big, big ones. So, the small once like the organic one that we have just mentioned no nobody will purchase that one. If you send 10 bags of cabbage and they are bigger ones, the hotels will buy all but reject the small ones. (FG, 4)

According to the farmers, consumers reject organic products for being tiny perhaps due to either limited knowledge of food production processes or desire to have enough food to feed the family. One of the participants asserts “Those who know when they go to the market, they will buy the tiny ones and leave the bigger ones. Some people are in Accra that have never seen farm before. . . So, some people don’t know” (Elizabeth). This calls for public health promotion education focusing on healthy eating, particularly emphasizing the characteristics and health benefits of organic foods. That said, few organic retail shops are located in the cities for more affluent people. Notably, the researchers observed that health-conscious farmers produce food organically for their own consumption and use agrochemicals for products that they intend to sell while other farmers consider such practice morally and ethically wrong as one of the participants asserts:

Me for instance, I don’t use fertilizer to grow maize. . . we apply it in excess with the expectation that we will get more yield. But don’t you know that it will affect your health? If you think you don’t mind because you are going to sell that is not good, because you are protecting your life, but you are destroying someone else life. So, me I don’t use fertilizer for cassava and maize. (Nii-Quaye)

Nii-Quaye advises farmers to consider their health and that of consumers in their farming practices. This is relevant advice as traces of agrochemicals have been found in some food products in Ghana.^62^ Another issue that emanates from competition among farmers is the distribution of risk and uncertainty. Because of small landholdings among the farmers in Ghana, farmers usually grow crops in clusters surrounded by other farmers. Therefore, if farmers in a cluster decide to engage in organic farming, they share the risk of pest infestation. However, if others use agrochemicals and 1 farmer decides to do organic farming, the pests from his neighbors’ farms will move to his farm and cause havoc. Furthermore, there is likelihood of contamination due to cross pollination. Therefore, farmers do not have the incentive to engage in organic farming due to the competition from other farmers.

## Chemical Use Practices and Safety Precautions Among Smallholder Farmers

The study discovered that although most of the farmers used agrochemicals, their knowledge in terms of safety precautions is limited as noted by previous studies.^5,12,70^ Furthermore, farmers fail to appreciate the health and environmental effects of agrochemicals. The major source of agrochemicals information to farmers comes from extension officers, agrochemical dealers, farmer colleagues, and the farmers’ own self-intuitions. This confirms the earlier studies in Ghana.^12,21,69^ However, the majority of the smallholder farmers do not have access to extension services^1,17^ and the majority of agrochemical dealers are not trained chemists.^12^ Hence, their advice is based on their personal experience, intuition, or sometimes conjecture. The findings contradict Mutune et al^63^ who reported that most smallholder farmers of Nyeri County in Kenya had received formal training on pesticide application. However, it supports Imoro et al^12^ who found that the majority (74.4%) of chemical attendants in the Tolon District of the Northern Region of Ghana were high school drop-outs and none of them had tertiary education, although this appears to depend on the geographic location. Indeed, Onwona-Kwakye et al^21^ found that the majority of farmers in their study received training on pesticide use in Ghana. Their study further found that farmers do not observe basic safety precautions such as re-entry period and most farmers spray pesticides against the wind, a finding that confirm the earlier study in Uganda.^14^

The present study also observed that farmers engage in dangerous practices, such as mixing fertilizer and herbicides, with the hope that the concoction can kill weeds and at the same time provide nutrients to their crops. The result, therefore, affirms the findings of Mattah et al^51^ who discovered that farmers used a cocktail of pesticides to control pests. Some smallholder farmers who called themselves professional sprayers even go to the extent of testing the concentration of the chemicals with their tongue. Others mix fertilizer and herbicide in barrels and allow the mixture to stay on the farm overnight, exposing the mixture to beneficial insects such as bees and other pollinators. Based on the farmers’ practices, it is evident that present agrochemical uses in Ghana pose an acute risk to the aquatic and terrestrial organisms.^12,21,64^ Additionally, these practices present eminent danger to beneficial insects such as bees, crop pollinators, and other soil fauna such as earthworms that help to aerate the soil. Also, sprayers submerge their knapsack sprayer in the barrel and place it on their back with some of the chemicals spilling over their bodies. All of these practices expose farmers to chemical poisoning and serious negative health effects. The study also exposes important gendered dimensions to these risks and associated adverse health consequences. Specifically, it found that men did most of the spraying compared to women. The study further observed that women farmers in the Northern belt lack financial resources to purchase agrochemicals compared to their male counterparts or women farmers in the Southern belt; hence, they rely on their Indigenous methods of pest management and manure application to produce African Indigenous leafy vegetables (AILVs). Ayishatu, a leader of women groups locally called “megagiya” in one of the communities in the Northern belt, asserts:

. . .we as women, we don’t have the resources to buy fertilizers like the men do. So, if you look at some of the vegetables I talked about, okro and those things, sometimes with such crops you will see termites try to chop and push some of this plant down. So, what we do is that we take the ash and mix it with animal droppings and go and broadcast on the farm, that way the termite will all go and the animal droppings will improve the fertility of the soil. (IdI)

This knowledge was lacking in the Southern belt. For instance, on one of the farms we visited, farmers had serious issues with termite infestation but were short of ideas as to how to deal with the situation. Similarly, the study found use of inorganic fertilizers was more common in the Northern belt than the Southern belt but at par with farmers in the Middle belt. Interaction with farmers also revealed the use of agrochemicals as a coping mechanism to adjust to climate change rather than as a convenience as found in other studies.^65^ Lastly, growing crops without agrochemicals is unfeasible in some communities in the Northern belt because of the adoption of short duration varieties.

## Health Implications

The study found two major health implications as reported by farmers: chemical poisoning and lower self-reported health. The subsequent sections discuss the health implications, starting with chemical poisoning.

### Chemical poisoning

Many of the study participants reported falling ill after applying agrochemicals but contend that they have no options. Alice confirmed experiencing chemical poisoning: “Yes, sometimes I fall sick after spraying but if I did not do it, I will not get anybody to do it for me.” Alice’s quote confirms the finding that the majority (80%) of vegetable farmers in Ghana become ill from chemical exposures and that the common symptoms of chemical poisoning are body weakness and headache/dizziness.^5,66^ Dzobo^50^ found that farmers complained about the following: body weakness (89.2%), sexual weakness (ie, impotence) among the men (24.5%), chronic cough (15.4%), and depression (14.3%) as symptoms of chemical poisoning. Similar findings have been reported in Uganda^14^ and Kenya.^63^

Study participants also lacked access to recommended protective gear: overall coats, nose masks, Wellington boots, and gloves. This confirms other findings that 74% of farmers spray chemicals without appropriate PPE, further exposing themselves to risk.^5,6^ It is, therefore, vital to study the correlation between the use of agrochemicals and cancer cases in rural Ghana as these diseases are staggering in communities that hitherto had no records of chronic illness.^68^ Furthermore, and equally concerning, some farmers admitted that their children help them in the spraying process and do so without wearing any protective clothing. The involvement of children in the application of these chemicals is an especially dangerous practice because their internal organs and immune systems are still developing. This vulnerability, in part, contributes to their significantly heightened risk of well known deleterious health and development effects of exposure to pesticides.

### Low self-reported health

Although most farmers claimed they are healthy because they consume the Indigenous food crops, some reported poor-health due to their chemical ingestion. Even those who argued they never visited a hospital conceded that their children or families fall sick regularly. Others reported loss of strength claiming they cannot work as effectively as before.

If you look at us sitting down here, we are supposed to be stronger than this, but we are not. If you get up in the morning if any member of your family is sick, it gives you a headache, as you are thinking you are depreciating, emaciating. . . (WS2)

As the above quote demonstrates, farmers in the study recognized that when they or their family members fall sick it undermines their physical and psychological wellbeing as well as their productivity.

## Conclusion

This study examined agrochemical use practices and the related health challenges of smallholder farmers in Ghana. The study sample was drawn from three agro-ecological zones out of Ghana’s seven agro-ecological zones. These agroecological zones were purposively selected to capture variation in climate change experiences, food production systems, and cultural practices among smallholder farmers in the agroecological zones. Through qualitative research methods, the study discovered five factors that compel/motivate farmers to use agrochemicals. First are environmental challenges such as erratic rainfall patterns, prolonged drought, and soil depletion which compel farmers to adopt agrochemicals. Second, are the activities of NGOs who introduced some farmers to short-duration varieties that are bred to adapt to agrochemicals. The third is the government fertilizer subsidy program introduced in 2008 to motivate farmers to use other agrochemicals such as herbicides and insecticides. Fourth, is the lack of or high cost of labor motivates farmers to use agrochemicals that they consider reliable and cost-effective. Finally, competition among smallholder farmers and consumer willingness to purchase conventional foods at the expense of organic foods compels farmers to use agrochemicals.

The study also revealed that farmers do not strictly observe safety precautions associated with the use of agrochemicals due to their limited knowledge of active ingredients of agrochemicals and the subsequent normalization of most agrochemicals. Furthermore, the study discovered chemical poisoning and low self-reported health as major implications of agrochemicals use within the communities. This study, therefore, recommends continuous and rigorous health promotion campaigns for both consumers and farmers to reduce the menace of agrochemical use in Ghana.

## References

[1] Demi SM. Assessing Indigenous Food Systems and Cultural Knowledges Among Smallholder Farmers in Ghana: Towards Environmental Sustainability Education and Development. Unpublished doctoral dissertation. University of Toronto; 2019.

[2] Tambo JA Kansiime MK Mugambi I, et al. Understanding smallholders’ responses to fall armyworm (Spodoptera frugiperda) invasion: evidence from five African countries. Sci Total Environ. 2020;740:1-2. 10.1016/j.scitotenv.2020.140015 32927537

[3] Williamson S Ball A Pretty J. Trends in pesticide use and drivers for safer pest management in four African countries. Crop Prot. 2008;27:1327-1334.

[4] European Parliament. The Use of Pesticides in Developing Countries and Their Impact on Health and the Right to Food. Policy Department for External Relations Directorate General for External Policies of the Union; 2021. https://www.europarl.europa.eu/cmsdata/219887/Pesticides%20health%20and%20food.pdf

[5] Ntow WJ Gijzen HJ Kelderman P Drechsel P. Farmer perceptions and pesticide use practices in vegetable production in Ghana. Pest Manag Sci. 2006;62:356-365. 16532443 10.1002/ps.1178

[6] Ikerd J. Family farms of North America. International Policy Centre for Inclusive Growth (IPC-IG) working paper 152. FAO and UNDP; 2016.

[7] MoFA [Ministry of Food and Agriculture]. Agricultural Sector Progress Report 2015. Government of Ghana; 2016. Agriculturalsectorprogressreport2015_Final.pdf.

[8] MoFA [Ministry of Food and Agriculture]. Agriculture in Ghana: Fact and Figures 2015. Government of Ghana; 2016. Agriculture-in-Ghana-Facts-and-Figures-2015.pdf.

[9] MoFA [Ministry of Food and Agriculture]. Medium Term Agricultural Sector Investment Plan (METASIP) II, 2014–2017. Ministry of Food and Agriculture; 2015. METASIPII2014-17.pdf.

[10] Lowder SK Skoet J Raney T. The number, size, and distribution of farms, smallholder farms, and family farms worldwide. World Dev. 2016;87:16-29.

[11] Afari-Sefa V Asare-Bediako E Kenyon L Micah JA. Pesticide use practices and perceptions of vegetable farmers in the cocoa belts of the Ashanti and western regions of Ghana. Adv Crop Sci Technol. 2015;3:174.

[12] Imoro ZA Larbi J Duwiejuah AB. Pesticide availability and usage by farmers in the northern region of Ghana. J Health Pollut. 2019;9:190906-190908. 31497369 10.5696/2156-9614-9.23.190906 PMC6711326

[13] Issahaku G Abdulai A. Sustainable land management practices and technical and environmental efficiency among smallholder farmers in Ghana. J Agric Appl Econ. 2020;52:96-116.

[14] Godfrey KH Patrick K Judith K, et al. Farmers’ knowledge and perception of the use of pesticides in Arabica coffee, Coffea arabica agro-ecologies of Uganda. J Agric Environ Sci. 2018;7:173-188.

[15] Ndayambaje B Amuguni H Coffin-Schmitt J Sibo N Ntawubizi M VanWormer E. Pesticide application practices and knowledge among small-scale local rice growers and communities in Rwanda: a cross-sectional study. Int J Environ Res Public Health. 2019;16:4770. 10.3390/ijerph16234770 PMC6926630 31795202

[16] Okonya JS Petsakos A Suarez V, et al. Pesticide use practices in root, tuber, and banana crops by smallholder farmers in Rwanda and Burundi. Int J Environ Res Public Health. 2019;16:400. 10.3390/ijerph16030400 PMC6388262 30708958

[17] Lee N. Assuring safe pesticide use for commercial crops, including soybean, in Sub-Saharan Africa. Afr J Food Agric Nutr Dev. 2020;19:15173-15176.

[18] Adekunle CP Akinbode SO Akerele D Oyekale TO Koyi OV. Effects of agricultural pesticide utilization on farmers health in Egbeda local government area, Oyo state, Nigeria. Niger J Agric Econ. 2017;7:73-88.

[19] Liverpool-Tasie LSO Omonona BT Sanou A Ogunleye WO. Is increasing inorganic fertilizer use for maize production in SSA a profitable proposition? Evidence from Nigeria. Food Policy. 2017;67:41-51. 28413245 10.1016/j.foodpol.2016.09.011 PMC5384440

[20] Sheahan M Barrett CB. Ten striking facts about agricultural input use in Sub-Saharan Africa. Food Policy. 2017;67:12-25. 28413243 10.1016/j.foodpol.2016.09.010 PMC5384438

[21] Onwona-Kwakye M Hogarh JN Van den Brink PJ. Environmental risk assessment of pesticides currently applied in Ghana. Chemosphere. 2020;254:126845. 32334242 10.1016/j.chemosphere.2020.126845

[22] Kariathi V Kassim N Kimanya M. Pesticide exposure from fresh tomatoes and its relationship with pesticide application practices in Meru district. Cogent Food Agric. 2016;2:1-12.

[23] Shashi K Marcella V. Cocoa in Ghana: shaping the success of an economy. In: Chuhan-Pole P Angwafo M, eds. Yes, African Can: Success Stories From a Dynamic Continent. World Bank; 2011.

[24] Adu P Forkuo EK Issah A, et al. High incidence of moderately reduced renal function and lead bioaccumulation in agricultural workers in Assin South district, Ghana: a community-based case-control study. Int J Nephrol. 2019;2019:1-7. 10.1155/2019/5368427 PMC6791189 31662908

[25] Environmental Protection Agency, Ghana. Annual Report, EPA; 2015.

[26] Vijver MG Hunting ER Nederstigt TAP Tamis WLM van den Brink PJ van Bodegom PM. Postregistration monitoring of pesticides is urgently required to protect ecosystems. Environ Toxicol Chem. 2017;36:860-865. 28370291 10.1002/etc.3721

[27] NPASP (Northern Presbyterian Agricultural Services and Partners). Ghana’s Pesticide Crisis: The Need for Further Government Action. NPASP; 2012:50. https://curtisresearch.org/ghanas-pesticide-crisis-the-need-for-further-government-action/

[28] Bezu S Kassie GT Shiferaw B Ricker-Gilbert J. Impact of improved maize adoption on welfare of farm households in Malawi: a panel data analysis. World Dev. 2014;59:120-131.

[29] Xiang H Wang YH Huang QQ Yang QY. How much is the eco-efficiency of agricultural production in West China? Evidence from the village level data. Int J Environ Res Public Health. 2020;17:4049-4115. 10.3390/ijerph17114049 PMC7311960 32517143

[30] Zahedi SM Karimi M Teixeira da Silva JA. The use of nanotechnology to increase quality and yield of fruit crops. J Sci Food Agric. 2020;100:25-31. 31471903 10.1002/jsfa.10004

[31] Danso-Abbeam G Baiyegunhi LJS. Adoption of agrochemical management practices among smallholder cocoa farmers in Ghana. Afr J Sci Technol Innov Dev. 2017;9:717-728.

[32] Harrison RD Thierfelder C Baudron F, et al. Agro-ecological options for fall armyworm (Spodoptera frugiperda JE Smith) management: providing low-cost, smallholder friendly solutions to an invasive pest. J Environ Manag. 2019;243:318-330. 10.1016/j.jenvman.2019.05.011 31102899

[33] Hruska AJ. Fall armyworm (Spodoptera frugiperda) management by smallholders. CAB Rev. 2019;14:1-11.

[34] Ali MP Kabir MMM Haque SS, et al. Farmer’s behavior in pesticide use: insights study from smallholder and intensive agricultural farms in Bangladesh. Sci Total Environ. 2020;747:747:1-20. 10.1016/j.scitotenv.2020.141160 32781314

[35] Chimweta M Nyakudya IW Jimu L Bray Mashingaidze A. Fall armyworm [Spodoptera frugiperda (JE Smith)] damage in maize: management options for flood-recession cropping smallholder farmers. Int J Pest Manag. 2020;66:142-154.

[36] Kansiime MK Mugambi I Rwomushana I, et al. Farmer perception of fall armyworm (Spodoptera frugiderda J.E. Smith) and farm-level management practices in Zambia. Pest Manag Sci. 2019;75:2840-2850. 31148397 10.1002/ps.5504 PMC6771660

[37] Kumela T Simiyu J Sisay B, et al. Farmers’ knowledge, perceptions, and management practices of the new invasive pest, fall armyworm (Spodoptera frugiperda) in Ethiopia and Kenya. Int J Pest Manag. 2019;65:1-9.

[38] Akutse KS Kimemia JW Ekesi S Khamis FM Ombura OL Subramanian S. Ovicidal effects of entomopathogenic fungal isolates on the invasive fall armyworm Spodoptera frugiperda (Lepidoptera: Noctuidae). J Appl Entomol. 2019;143:626-634.

[39] Zaitzove-Raz M Comay O Motro Y Dayan T. Barn owls as biological control agents: potential risks to non-target rare and endangered species. Anim Conserv. 2020;23:646-659.

[40] Ghana Statistic Service. Ghana Living Standard Survey Round 6 (GLSS6), Main Report. Ghana Statistical Service; 2014.

[41] Poku A-G Birner R Gupta S. Making contract farming arrangements work in Africa’s bioeconomy: evidence from Cassava outgrower schemes in Ghana. Sustainability. 2018;10:1604-1621.

[42] Tonah S. The presidential special initiative on cassava: a bane or blessing to Ghana’s smallholder farmers. Ghana J Dev Stud. 2006;3:66-84.

[43] Freeman T. Best practice’ in focus group research: making sense of different views. J Adv Nurs. 2006;56:491-497. 17078825 10.1111/j.1365-2648.2006.04043.x

[44] Muijeen K Kongvattananon P Somprasert C. The key success factors in focus group discussions with the elderly for novice researchers: a review. Health Res J. 2020;34:359-371.

[45] Appiah-Opoku S. Indigenous economic institutions and ecological knowledge: a Ghanaian case study. Environmentalist. 1999;19:217-227.

[46] Bhattarai B Beilin R Ford R. Gender, agrobiodiversity, and climate change: a study of adaptation practices in the Nepal Himalayas. World Dev. 2015;70:122-132.

[47] Maxwell JA. Qualitative Research Design: An Interactive Approach. Sage; 2012.

[48] Palinkas LA Horwitz SM Green CA Wisdom JP Duan N Hoagwood K. Purposeful sampling for qualitative data collection and analysis in mixed method implementation research. Adm Policy Ment Health. 2015;42:533-544. 24193818 10.1007/s10488-013-0528-y PMC4012002

[49] Anang BT Amikuzuno A. Factors influencing pesticide use in smallholder rice production in Northern Ghana. Agric For Fish. 2015;4:77-82.

[50] Dzobo A. Knowledge, Practices and Self-Reported Symptoms of Pesticides Use Among Vegetable Farmers: A Cross Sectional Study in the Offinso North District. Unpublished, Masters dissertation Submitted to School of Public Health. University of Ghana; 2016.

[51] Mattah MM Mattah PA Futagbi G. Pesticide application among farmers in the catchment of Ashaiman irrigation scheme of Ghana: health implications. J Environ Public Health. 2015;2015:1-7. 10.1155/2015/547272 PMC4698959 26798369

[52] Dinham B. Growing vegetables in developing countries for local urban populations and export markets: problems confronting small-scale producers. Pest Manag Sci. 2003;59:575-582. 12741526 10.1002/ps.654

[53] Avea A Zhu J Tian X, et al. Do NGOs and development agencies contribute to sustainability of smallholder soybean farmers in Northern Ghana—a stochastic production frontier approach. Sustainability. 2016;8:465.

[54] Osei G. Self-help without the self: critique of non-governmental organizational approaches to rural development in Ghana. Int Soc Work. 2017;60:494-506.

[55] Scown MW Brady MV Nicholas KA. Billions in misspent EU agricultural subsidies could support the sustainable development goals. One Earth. 2020;3:237-250. 34173533 10.1016/j.oneear.2020.07.011 PMC7441947

[56] Jepson PC Guzy M Blaustein K, et al. Measuring pesticide ecological and health risks in West African agriculture to establish an enabling environment for sustainable intensification. Phil Trans R Soc A. 2014;369:20130491. 10.1098/rstb.2013.0491 PMC3928896 24535399

[57] Matthews G Wiles T Baleguel P. A survey of pesticide application in Cameroon. Crop Prot. 2003;22:707-714.

[58] Lencucha R Pal NE Appau A Thow A-M Drope J. Government policy and agricultural production: a scoping review to inform research and policy on healthy agricultural commodities. Global Health. 2020;16:11-15. 31959213 10.1186/s12992-020-0542-2 PMC6971899

[59] Łuczka W Kalinowski S. Barriers to the development of organic farming: a polish case study. Agriculture. 2020;10:536.

[60] Demi SM. Local Ghanaians’ resistance against GM crops. In: Dei GJS Demi SM, eds. Theorizing the ‘Anti-Colonial’. DIO Press Inc; 2021:125-139.

[61] Rock J. “We are not starving”: challenging genetically modified seeds and development in Ghana. Cult Agric Food Environ. 2019;41:15-23.

[62] Abagale SA Atiemob S Abagale FK, et al. Pesticide residues detected in selected crops, fish, and soil from irrigation sites in the Upper East region of Ghana. Adv J Chem Sect A. 2020;3:221-236.

[63] Mutune B Gachohi J Kikuvi G Niassy S Bii C. Knowledge and practices of pesticides used against the bean fly (Ophiomyia phaseoli) and associated health effects among bean (Phaseolus vulgaris) smallholder farmers in Kabaru location, Nyeri County. Int J Health Sci. 2018;6:77-89.

[64] Frimpong JO Ofori ES Yeboah S, et al. Evaluating the impact of synthetic herbicides on soil dwelling macrobes and the physical state of soil in an agro-ecosystem. Ecotoxicol Environ Saf. 2018;156:205-215. 29550438 10.1016/j.ecoenv.2018.03.034

[65] Ahmed A Lawson ET Mensah A Gordon C Padgham J. Adaptation to climate change or non-climatic stressors in semi-arid regions? Evidence of gender differentiation in three agrarian districts of Ghana. Environ Dev. 2016;20:45-58.

[66] Dewi VS Rivai T. The behavior of pesticide usage and risk of health disorders in vegetable farmers. J Phys Conf Ser. 2019;1244:1-6.

[67] Oyekale AS. Cocoa farmers’ safety perception and compliance with precautions in the use of pesticides in centre and Western Cameroon. Appl Ecol Environ Res. 2017;15:205-219.

[68] de-Graft Aikins A Addo J Ofei F Bosu W Agyemang C. Ghana’s burden of chronic non-communicable diseases: future directions in research, practice, and policy. Ghana Med J. 2012;46:1-3. 23661810 PMC3645141

[69] Obiri BD Obeng EA Oduro KA, et al. Farmers’ perceptions of herbicide usage in forest landscape restoration programs in Ghana. Sci Afr. 2021;11:1-16.

[70] Wumbei A Houbraken M Spanoghe P. Pesticides use and exposure among yam farmers in the Nanumba traditional area of Ghana. Environ Monit Assess. 2019;191:1-16. 31028501 10.1007/s10661-019-7449-5

[71] Oesterlund AH Thomsen JF Sekimpi DK Maziina J Racheal A Jørs E. Pesticide knowledge, practice and attitude and how it affects the health of small-scale farmers in Uganda: a cross-sectional study. Afr Health Sci. 2014;14:420-433. 25320593 10.4314/ahs.v14i2.19 PMC4196420

[72] Chow L. Glyphosate Exposure Increases Cancers Risk by up to 41% Study Finds. Ecowatch; 2019. https://www.ecowatch.com/glyphosate-cancer-2628948966-2628948966.html

[73] Cohen P. Roundup maker to pay $10 billion to settle cancer suits. The New York Times. https://www.nytimes.com/2020/06/24/business/roundup-settlement-lawsuits.html

[74] Danquah OA Ekor AK Asuming-Brempong S. Insecticide use pattern on tomatoes produced at Yonso community in the Sekyere West district of Ashanti region, Ghana. Ghana J Agric Sci. 2010;42:55-63.

[75] Fianko JR Donkor A Lowor ST Yeboah PO. Agrochemicals and the Ghanaian environment, a review. J Environ Prot. 2011;2:221-230.

